# Beat-based dancing to music has evolutionary foundations in advanced vocal learning

**DOI:** 10.1186/s12868-024-00843-6

**Published:** 2024-11-06

**Authors:** Aniruddh D. Patel

**Affiliations:** 1 Department of Psychology, Tufts University, 490 Boston Ave., Medford, MA 02155 USA; 2 Program in Brain, Mind, and Consciousness, Canadian Institute for Advanced Research, Toronto, Canada

**Keywords:** Rhythm, Evolution, Brain, Dance, Vocal learning, Parietal cortex, Synchrony, Speech

## Abstract

Dancing to music is ancient and widespread in human cultures. While dance shows great cultural diversity, it often involves nonvocal rhythmic movements synchronized to musical beats in a predictive and tempo-flexible manner. To date, the only nonhuman animals known to spontaneously move to music in this way are parrots. This paper proposes that human-parrot similarities in movement to music and in the neurobiology of advanced vocal learning hold clues to the evolutionary foundations of human dance. The proposal draws on recent research on the neurobiology of parrot vocal learning by Jarvis and colleagues and on a recent cortical model for speech motor control by Hickock and colleagues. These two lines of work are synthesized to suggest that gene regulation changes associated with the evolution of a dorsal laryngeal pitch control pathway in ancestral humans fortuitously strengthened auditory-parietal cortical connections that support beat-based rhythmic processing. More generally, the proposal aims to explain how and why the evolution of strong forebrain auditory-motor integration in the service of learned *vocal* control led to a capacity and proclivity to synchronize *nonvocal* movements to the beat. The proposal specifies cortical brain pathways implicated in the origins of human beat-based dancing and leads to testable predictions and suggestions for future research.

## Background

Dance occurs in human societies around the world and is intimately related to music. While dance movements vary widely across cultures and eras, rhythmic coordination of such movements with musical beats is commonly observed [1, 2]. Rhythmic movement to beat-based music often emerges spontaneously in infancy or early childhood. (Throughout this paper “spontaneously” means “without reliance on formal training”, not “without reliance on social interaction”). At this age movements are not synchronized to beats, although movements can exhibit tempo flexibility, i.e., faster movements to faster-tempo music [3–5]. Beat-synchronized movement emerges spontaneously over the first decade of life [6]. Such synchronization is *predictive* and *tempo flexible*. *Predictive* means that rhythmic movements anticipate the beat with a high degree of temporal precision, as shown by the fact that people often bob, clap, or step very close to the time of beats, and often slightly ahead of the beat. *Tempo flexible* means that such predictive synchronization is maintained over a wide range of tempi. For example, one study found that Western dance music ranged from 94 to 176 beats per minute (BPM) [7], meaning that people readily synchronize to beats across a tempo range of ± 30% relative to the middle of this range (135 BPM).

Some people struggle with musical beat synchronization, likely due to a combination of experiential and genetic factors [8, 9], and the ability is enhanced by music or dance training [10]. However, a large majority of adults have this ability [9, 11]. When used in social situations such as group dancing and singing, the ability allows multiple individuals to synchronize rhythmic movements and/or sounds with each other, a collective behavior with measurable psychological and social consequences [12] that may have benefited human ancestors over the course of human evolution [13–15].

Humans are not the only dancing species. A number of non-human animals have behaviors that biologists call dance. Many examples come from birds: one empirically-studied case is the multimodal courtship dance of male lyrebirds [16]. What can we learn about the evolution of human dance from cross-species research on dancing? One approach is to focus on *homology*, i.e., similar traits inherited from a common ancestor, and on *convergence*, i.e., similar traits arising independently in separate lineages. In terms of homology, it is notable that chimpanzees (who along with bonobos are our closest living relatives) sometimes produce a rhythmic “rain dance” in the wild in response to loud sounds such as thunder, rain, or waterfalls [17, 18]. While such sounds are not beat-based, laboratory experiments show that complex beat-based rhythms can elicit spontaneous swaying, clapping, or other rhythmic movements in adult chimpanzees, with faster auditory rhythms eliciting faster rhythmic movement [19, 20]. However, such movements are not synchronized to beats and occur even when the rhythms are scrambled and lack an underlying beat. Given the small number of animals studied in this research, more research is needed in order to understand how rhythmic movements in chimpanzees are related to the structure of complex sound patterns. Such research could help suggest which precursors to dance were present in the last common ancestor of humans and chimpanzees around 7 million years ago.

Turning from homology to convergence, a key issue is which species can be meaningfully compared to humans in terms of dance. The similarities between human and nonhuman dance are a topic of current interest and debate [21]. Interestingly, dance seems to be far more common in the natural behavior of birds than of mammals [22–24], making avian behavior a rich resource for studies of the convergently evolved features of human and animal dance. The current paper focuses on a group of birds that makes dance-like movements to human music, namely parrots.

When raised by humans and exposed to beat-based music, some parrots spontaneously synchronize their movements to musical beats in a predictive and tempo flexible manner [25, 26]. To be clear, such parrots do not rival adult humans in their synchronization abilities. A male sulphur-crested cockatoo named Snowball, for example, synchronized his head bobs predictively to musical beats in episodic “bouts” (median = 16 head bobs/bout) interspersed in longer stretches of unsynchronized rhythmic movement during which he gravitated to a head bob tempo near 126 BPM [25, 27]. When the original song Snowball danced to was sped up or slowed down, Snowball exhibited this episodic synchrony at 9 different tempi ranging from 10% slower to 20% faster than the original song (108.7 BPM) [25]. During synchronized bouts head bobs were often closely aligned to beats (averaging near 0° phase difference), akin to the pattern of human synchronization. Snowball’s episodic synchronization, however, differs from the abilities of human adults, who can sustain predictive synchrony at various tempi for much longer periods, e.g., over an entire song. Critically, statistical analysis (a permutation test using Monte Carlo methods) showed that the amount of predictive synchrony Snowball showed across tempi was highly unlikely to be due to chance alignment of rhythmic head bobs to beats. Schachner and colleagues also applied statistical methods to show significant predictive synchronization of rhythmic movement to musical beats in males and females of several other parrot species [26, 28].

The sporadic synchronization seen in Snowball may be more akin to the abilities of human children rather than adults. Recall that infants and young children moving rhythmically to music do not synchronize to beats, yet most adults do. It seems unlikely that children go through a binary transition from no synchronization abilities to full adult-like abilities. There may be a developmental period in which children can synchronize predictively across a range of tempi but show sporadic synchrony interspersed with unsynchronized rhythmic movements that tend toward a preferred tempo [5]. Further work on the ontogeny of beat-based movement to music in humans is needed to determine if this is the case. Ontogenetic work on rhythmic movement to music is also needed in parrots. At present we do not know what proportion of pet parrots raised around beat-based music develop this behavior, nor what characteristics of individual parrots or their rearing experiences enhance the likelihood of the behavior. Also, it is not yet known if this behavior only develops in certain parrot species. Spontaneous rhythmic movement to music has not been documented in pet budgerigars, for example, although lab studies show they can synchronize pecks to audio-visual metronomes [29, 30]. Thus while many questions remain about parrot movement to music, when the behavior emerges the resemblance to human behavior can be striking. For example, some parrots develop a rich diversity of dance-like movements in response to beat-based music despite no formal training to do so (e.g., foot lifts coordinated with head swings, headbanging, etc.; see videos in [31]).

The similarities between parrot and human movement to music are surprising given that parrots are more closely related to extinct dinosaurs than they are to humans. The last common ancestor of humans and birds lived around 320 million years ago. In sharp contrast, chimpanzees and bonobos shared a common ancestor with humans about 7 million years ago, and their brains are much closer to ours in size and gross anatomy than are avian brains. Yet spontaneous predictive and tempo-flexible synchronization to a musical beat has never been reported in chimpanzees or bonobos, despite numerous cases of these animals being raised by humans and spontaneously imitating human movements [32–34]. (Macaque monkeys can be trained to predictively synchronize movements to metronomes [35, 36], and research on monkey tapping to metronomes has provided valuable insights into the brain mechanisms of sensorimotor synchronization [37], but monkeys do not spontaneously engage in beat-synchronized movements to music.) Interestingly, beat synchronization has also not been observed in other pet animals which develop with exposure to human music and dance, such as dogs [26, 28]. This is true despite the facts that dogs are highly attentive to their human partners and can learn elaborate motor routines, including dance-like routines in the sport called “canine freestyle” [38].

To my knowledge, parrots are the only nonhuman animals who spontaneously move rhythmically to beat-based music in a predictive and tempo flexible manner.^1^ Why might this be the case? I propose that parrots’ vocal learning abilities are a key factor. More specifically, I argue that their advanced vocal learning system has neuroanatomical parallels to the human vocal learning system, and that the evolution of these systems fortuitously enabled a capacity and proclivity for human-like beat synchronization. (Throughout this paper “fortuitous” means “as a byproduct rather than due to natural selection for an ability”, rather than “lucky or fortunate”.) The fortuitous nature of this trait is clear in parrots, since moving in time with beat-based rhythms is not known to be part of their natural behavior, which effectively rules out any functional or adaptive significance. In humans, I have recently argued that natural selection elaborated this fortuitous trait in ancestral humans into a neural specialization for beat-synchronized movement to rhythms [41]. The proposed mechanism behind this process of natural selection was gene-culture coevolution. The current paper, however, aims to explain the fortuitous origins of beat synchronization abilities in parrots and ancestral humans, addressing how this capacity emerged in the first place. Thus this paper does not rely on the notion of subsequent evolutionary neural specialization for this trait, although I touch on this idea later.

While this paper proposes a link between musical beat synchronization and advanced vocal learning, it is important to note that animals can evolve rhythm processing abilities via different neural mechanisms. Thus I emphasize that I am not proposing that advanced vocal learning is the evolutionary foundation for all forms of dance, temporal coordination (e.g., vocal turn-taking), predictive timing, or rhythm perception, as these abilities are well documented in animals without advanced vocal learning [23, 42–47]. Instead, the focus is on a link between advanced vocal learning and a specific form of beat synchronization, as detailed below.

## Main text

### Relationship to earlier hypotheses

The current proposal builds on an earlier “vocal learning and rhythmic synchronization hypothesis” from 2006 linking complex vocal learning to the capacity for “beat perception and synchronization” or BPS, which refers to predictive and tempo-flexible motor synchronization to beats perceived in complex auditory stimuli such as music [48]. The hypothesis is thus not concerned with synchronization to relatively simple metronome-like signals, as seen in certain insects (e.g., crickets chirping together in synchrony [49–51]). In complex vocal learning, auditory input is needed to form templates which guide the development of the animal’s own vocalizations [52]. Complex vocal learning also involves temporally-precise online auditory-motor interactions in order to control anatomical structures, e.g., using auditory feedback to perceive and adjust vocal output [53, 54]. Complex vocal learning is a relatively rare trait in animals, found in songbirds, parrots, and hummingbirds, as well as in elephants and in some species of bats, cetaceans, and pinnipeds [55]. More species with this ability may come to light as research progresses, but among primates it appears that humans are the only species with this trait. Complex vocal learning can be distinguished from limited vocal learning, where the latter is “the ability to fine-tune acoustic features of species-specific vocalizations that can develop in the absence of auditory input because innate motor programs can generate the species-specific pattern” [56]. Unlike complex vocal learning, limited vocal learning may be widespread in nonhuman primates and other mammals, akin to the widespread capacity of mammals to exhibit learning that influences the usage and comprehension of sounds [57]. It has been theorized that limited and complex vocal learning lie along a continuum of animal vocal learning capacities [55].

Why would complex vocal learning have anything to do with BPS? Both involve strong integration between forebrain auditory and motor processing mechanisms. Like complex vocal learning, BPS relies on auditory-guided motor learning and precise online auditory-motor integration, both of which are required for predictive and tempo-flexible synchronization of movement to beats perceived in complex sound patterns. Notably, even though rhythmic patterns in the real world are often multimodal [58], human beat synchronization is far superior to complex auditory rhythms than to identical rhythms presented as visual or tactile patterns, suggesting that human BPS, like complex vocal learning, involves specialized auditory-motor processing [59, 60].

**Fig. 1:**
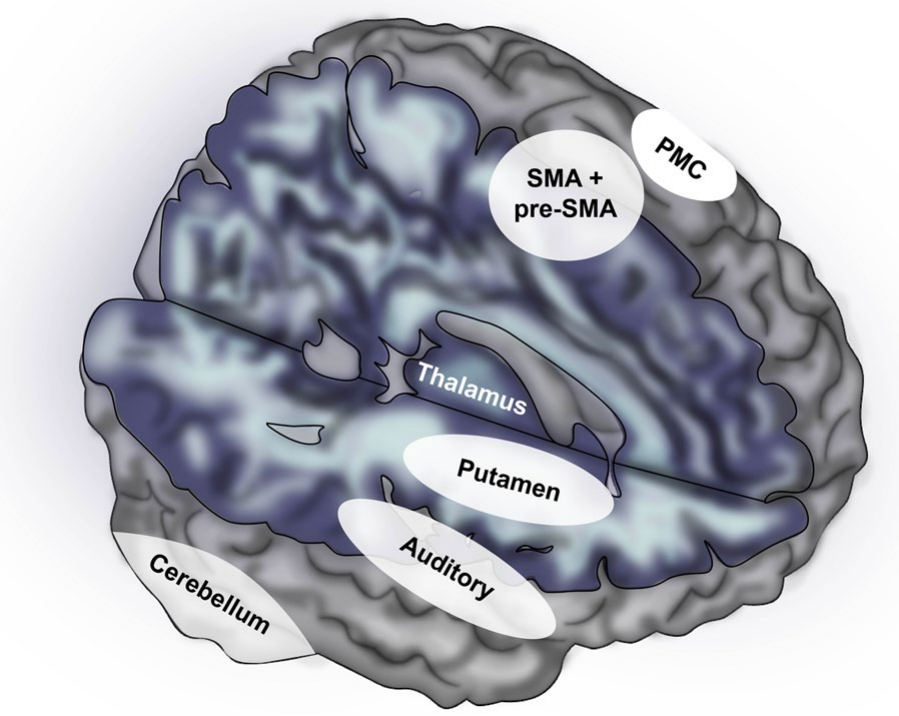
Schematic of key human auditory and motor brain regions involved in auditory beat-based processing. White circles or ovals with labels show general location of these areas as revealed by fMRI on a standard MRI model of the human brain. Premotor regions include supplementary motor area and pre-supplementary motor area (SMA and pre-SMA) and dorsal premotor cortex (PMC). The putamen is a region of the basal ganglia involved in motor control and is linked to premotor regions via a cortico-basal-ganglia-thalamic loop [66]. Current evidence suggests that basal ganglia regions play a more central role in beat-based timing than do cerebellar regions, which may be more important for single-interval timing [67, 68]. Note that some areas involved in beat processing, such as regions of the inferior parietal lobe, are not shown. For a recent review and metanalysis of neuroimaging studies of beat processing, see [69]

Complex vocal learning and BPS also have similarities in terms of their neural substrates. Research on related species with vs. without complex vocal learning (such as songbirds vs. chickens) has revealed neural specializations for vocal learning in premotor and basal ganglia regions connected to forebrain auditory regions (e.g., in songbirds, premotor area HVC and basal ganglia region Area X) [52, 55, 61, 62]. BPS also involves interactions between premotor, basal ganglia, and auditory forebrain regions [63, 64]. An interesting finding of human fMRI research is that during beat perception *even in the absence of movement* motor planning and basal ganglia regions are strongly active and interact with each other and with cortical auditory regions [65, 66] (Fig. 1). Several researchers theorize that this motor activity plays a role in predicting the timing of beats [66]. The motor system is adept at generating periodic movements on the timescale of beats (e.g., motor periodicities in human walking occur at rates of 80–150 BPM [70]). Thus motor planning regions are well-positioned to create periodic patterns of neural activity which can act as predictive signals for auditory regions to which the motor planning regions are reciprocally connected [71].

To summarize, the vocal learning and rhythmic synchronization hypothesis was motivated by a combination of behavioral, cross-species, and neural research. A prediction of the hypothesis is that only species with complex vocal learning are capable of BPS. Patel (2021) recently reviewed primate, avian, pinniped, and rodent research relevant to this prediction [41]. The hypothesis has been supported by several studies but has also faced some challenges, though I do not believe these are insurmountable (Box 1).

### Box 1: Challenges to the vocal learning and rhythmic synchronization hypothesis

**Table:** 

The vocal learning and rhythmic synchronization hypothesis (VLRSH) [48] would be challenged by evidence that a vocal non-learning species can synchronize predictively to musical beats and can generalize this ability across a broad range of tempi. An important study by Cook, Rouse and colleagues [72] used operant conditioning with food rewards to train a California sea lion named “Ronan” to synchronize her head bobs with the beat of a disco funk song. The researchers showed that this ability generalized when the song was presented at five novel tempi ranging from 20% slower to 10% faster than the original song (130 BPM). This challenged the VLRSH because this species is considered vocally inflexible. However, the vocal learning capacities of California sea lions have not been studied using modern methods which reveal remarkable vocal flexibility in other pinnipeds such as gray seals [73]. Also, as noted by others [74], the way Ronan synchronized her movements to musical beats was unlike human BPS in an important respect. When presented with the song at novel tempi Ronan’s head bobs lagged considerably behind the beat at the fastest tempo (~ 90^o^ phase difference on average) and occurred considerably before the beat at the slowest tempo (~ 60° phase difference on average) (Fig. 5 in [72]). This is unlike human BPS, in which rhythmic movements are much more consistently phase-aligned to beats across a comparable range of tempo variation (averaging close to 0° phase difference at each tempo). Ronan’s pattern of tempo-dependent phase leads and lags is reminiscent of an oscillator with a single intrinsic period driven by nearby frequencies [75], so her mechanisms of sync may be different from those involved in human-like BPS (see [41] for further discussion). Thus while this excellent study challenged the VLRSH, I believe it is premature to reject the hypothesis on the basis of these findings. Notably, this pioneering work helped paved the way for research on rhythmic processing in pinnipeds [76, 77], which is yielding new insights into relations between vocal learning and rhythmic capacities. More recently, a study of rats passively exposed to music at different tempi reported small head movements around the times of beats [78]. Initially this seemed to challenge the VLRSH since rats are vocal non-learners. However, as noted by other researchers [79], the small size of these movements, their tendency to immediately follow rather than precede beats, and the fact that they were only seen in the first experimental session suggest involuntary startle responses. Similar subtle facial movements in response to rhythmic sounds have recently been reported in mice (Fig. 1C in [80]). These reflexive motor reactions are distinct from the voluntary predictive movements involved in BPS, and thus do not challenge the VLRSH. Fortunately a growing body of research on motor synchronization to auditory rhythms in rodents focuses on voluntary movements [79, 81]. So far this work has used metronomes rather than complex sound patterns, so it remains to be seen if rodent research will challenge the VLRSH.	

Patel [41] also proposed a revised version of the vocal learning and rhythmic synchronization hypothesis aimed at explaining why BPS develops spontaneously in pet parrots but not in other vocal learning animals exposed to beat-based music, such as pet songbirds. The revised hypothesis built on neurobiological work showing that parrots have a more complex vocal learning system than songbirds [82, 83]. The 2021 paper also noted neuroanatomical parallels between the parrot vocal learning system and an influential model of cortical speech processing proposed by Hickok and Poeppel in 2007 [84]. The current paper expands on these ideas, drawing on a new update to this speech processing model by Hickok and colleagues [85].

More specifically, the current paper aims to explain the evolutionary foundations of human beat-based dancing to music by 1) examining similarities between parrot and human vocal learning systems and 2) suggesting how the evolution of these systems fortuitously gave rise to the capacity for BPS. To this end, the next section of the paper discusses parallels between parrot and human vocal learning systems. The subsequent section discusses why the evolution of these systems, which link complex auditory and *vocal* motor processing, would lead to rhythmic movement to beat-based music, which often links complex auditory and *nonvocal* motor processing. Finally, I suggest why the evolution of vocal learning in parrots and ancestral humans led not only to the capacity for BPS, but also to a spontaneous tendency to engage in this behavior, and I suggest why this tendency is not seen in other animals whose vocal learning skills rival those of pet parrots.

### Parallels between parrot and human vocal learning systems

**Fig. 2:**
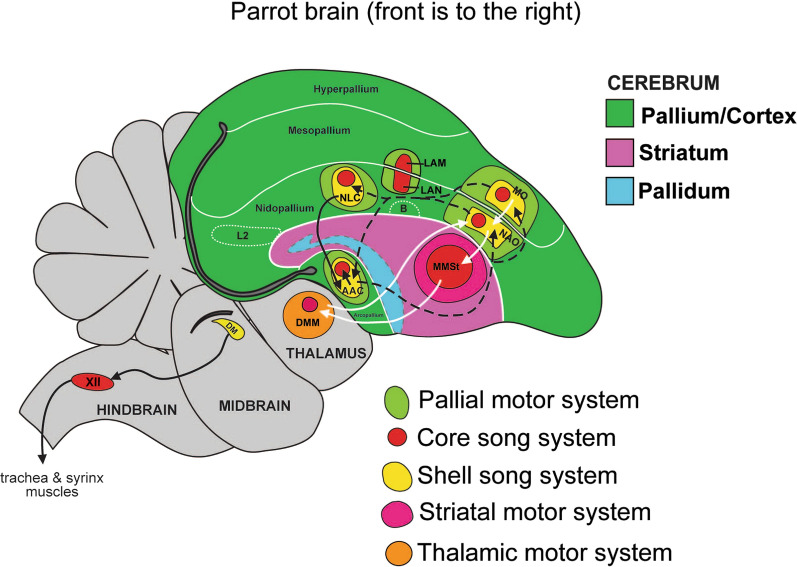
Schematic of core and shell systems for vocal learning in a parrot brain [83]. Red regions: core song system (similar to songbirds); yellow regions in pallium/cortex: shell song system (unique to parrots). Solid black arrow: shell system posterior vocal motor pathway; solid white arrows: shell system anterior vocal motor pathway; dashed arrows: connections between core and shell systems. For simplicity several connections are not shown, including connections among core system regions. Also not shown is the direct projection from AAC (central nucleus of the anterior arcopallium) core region to the brainstem’s hypoglossal nucleus (labelled XII in the figure’s hindbrain region): this projection is analogous to the direct projection in songbirds from the forebrain primary motor region RA (robust nucleus of the arcopallium) to XII. (See [83] for definitions of other acronyms.)

Like songbirds and humans, parrots actively control both a voice source and the geometry of their vocal tract to shape the acoustics of their learned vocalizations [86–91]. However, parrots have a more neurobiologically elaborate vocal learning system than songbirds, likely supporting their advanced vocal learning skills, which include the ability to imitate human speech and other complex sounds with high fidelity. The elaborate vocal learning circuitry of parrots was demonstrated by Jarvis and colleagues in neural research using gene expression and tract tracing to compare parrot brains from several species to the brain of a well-studied songbird species (the zebra finch) [82]. This work revealed that parrots not only have a premotor-motor-basal-ganglia-thalamic “core” system similar to songbirds, which controls the syrinx (the avian sound source, analogous to the mammalian larynx), they also have a “shell”’ system surrounding the core system, creating dual pathways for vocal learning and control (Fig. 2). At present there do not appear to be major differences between the core and shell system anatomy in male and female parrots [82], which aligns with the fact that both sexes have advanced vocal learning skills throughout life [92].

While the precise contributions of the core and shell pathways to parrot vocal learning and control are unclear, Patel (2021) [41] suggested that this two-pathway system might facilitate independent control of the syrinx and tongue during vocalization. Research indicates that parrots control movements in both structures to make vocal sounds. While songbirds can make some lingual movements to shape learned sounds (e.g., raising the tongue to tune vocal tract resonances to frequencies produced by the syrinx [87]), parrots may produce more sophisticated tongue movements during vocalization. Measurements reveal that parrots control both the height and front-back position of the tongue when producing learned sounds, and that these movements have acoustic consequences [88–90]. This lingual dexterity in the service of vocalization may stem from the fact that unlike songbirds, who tend to have blade-like tongues, parrots have a muscular prehensile tongue which manipulates nuts, fruit, or other items when feeding [93]. This high degree of tongue mobility may have been co-opted for vocalization in the evolution of the parrot lineage.

Intriguingly, there is a potential neural pathway between forebrain regions in the parrot shell system and tongue-controlling regions in the brainstem’s hypoglossal nucleus (region XII in Fig. 2): namely, projections from AAC shell to AAC core, and projections from AAC core to the lingual parts of the hypoglossal nucleus. (In songbirds the forebrain motor region RA, which is analogous to the AAC core in parrots, only projects to the syringeal part of the hypoglossal nucleus [62], whereas in parrots the AAC core projects to both lingual and syringeal parts of this nucleus, E. Jarvis, personal communication). Thus the shell system may allow greater control of the tongue during vocalization than possible with just the core system. Indeed, enhanced tongue control during vocalization may be essential to the complex acoustics of parrot vocalizations [94], since parrots, like humans, have just a single sound source, in contrast to songbirds who have two sources. This is because the parrot syrinx, like the human larynx, has vibrating membranes above the splitting of the bronchi from the trachea, whereas songbirds have separate syringeal membranes below this split, within each of their two bronchi [95].

Taking a step back, why would these details of the parrot vocal system be relevant to the current paper’s concerns? Chakraborty and Jarvis [83] proposed that the evolution of the shell system in parrots enhanced forebrain auditory-motor integration compared to songbirds (e.g., via more neural connections between forebrain auditory and motor regions) and suggested that this relates to parrots’ abilities to synchronize body movements to musical rhythms. I expand on this idea in the next section, after discussing a recent model for the neural control of speech. This model, like the parrot vocal learning system, involves two distinct pathways for controlling the acoustics of learned vocalizations.

**Fig. 3:**
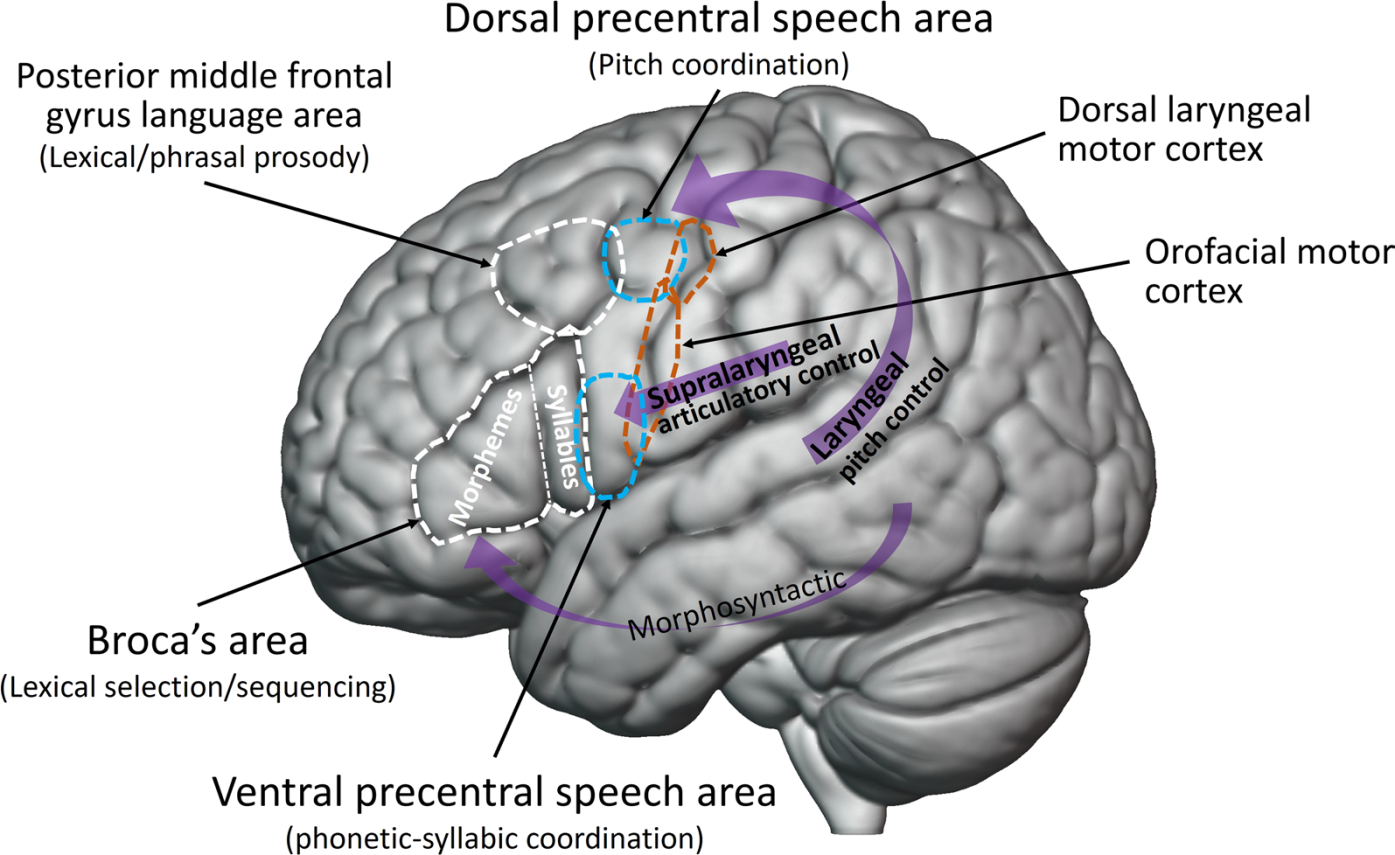
Schematic depiction of the dual speech coordination system model of Hickok and colleagues [85]. The laryngeal pitch control system is schematically represented via the upper curved purple arrow, and the supralaryngeal articulatory control system is schematically represented via the straight purple arrow (see text for details). The premotor dorsal precentral speech area involved in laryngeal pitch control is indicated by the upper dashed blue circle, above the dashed blue oval indicating the premotor ventral precentral speech area involved in the supralaryngeal articulatory control system. The dorsal laryngeal motor cortex and orofacial motor cortex are indicated by dashed orange ovals, with the location of the latter based on a meta-analysis from Guenther [96]. Frontal brain regions enclosed by white dashed lines and the brain system indicated by the curved purple arrow labelled “Morphosyntactic” are important for language and are discussed in Hickok et al. 2023 [85] but are not relevant to the current paper

Hickok and colleagues recently proposed a “dual speech coordination model” [85] which updates Hickok and Poeppel’s 2007 model of the cortical organization of speech processing [84]. According to the new model, which is based on an extensive review of empirical research, one neural system is involved in the laryngeal control of pitch while a second “supralaryngeal” system is primarily involved in coordinating articulators above the larynx (including the tongue) at the phonetic/syllabic level. Figure 3 shows these two systems schematically: the laryngeal pitch control system is represented by the upper curved purple arrow and the supralaryngeal articulatory system is represented by the straight purple arrow. In Hickok et al.’s new model [85] different types of input get more heavily weighted by each of the two systems: auditory input for the pitch control system and somatosensory input for the phonetic/syllabic system. (Note that the purple arrows in this figure represent functional systems, not specific white matter pathways: see [85] for neuroanatomical details). The laryngeal pitch control system connects auditory cortex to a dorsal premotor region, labelled “Dorsal precentral speech area” in Fig. 3, which is rostral to the dorsal laryngeal motor cortex. The supralaryngeal system includes a ventral premotor region, labelled “Ventral precentral speech area” in Fig. 3. While this ventral region may play a role in controlling the onset and offset of voicing (i.e., vocal fold adduction/abduction), intracranial recordings in humans indicate that a dorsal laryngeal control region supports volitional and precise control of vocal pitch (vocal fold tension) in speech and song [97]. It is currently unclear if the laryngeal pitch control system linking auditory and dorsal premotor regions is uniquely human among primates or is just much more strongly developed in humans than in primates with some degree of voluntary pitch control, such as marmosets [98–100].

From the perspective of the current paper, the laryngeal pitch control system in the dual speech coordination model is of interest because it involves strong coupling between auditory cortical regions (in Heschl’s gyrus and in higher-order regions beyond primary auditory cortex) and a dorsal premotor region. This premotor pitch control region is the “dorsal precentral speech area” represented by the upper dashed blue circle in Fig. 2. Recall that fMRI research shows that beat-based rhythmic processing involves strong coupling between auditory cortical and dorsal premotor regions [63–65] which are near the dorsal precentral speech area. For both laryngeal pitch control and beat processing, one line of evidence for strong auditory-motor coupling comes from the fact that the implicated dorsal premotor regions respond to auditory input in the absence of overt movement. Recall that fMRI research on beat perception in the absence of movement reveals strong activity in dorsal motor planning regions (e.g., SMA and PMC in Fig. 1). In the case of the laryngeal pitch control pathway, fMRI research reveals that the dorsal precentral speech area has speech-related spectrotemporal receptive field (STRF) properties, including tuning to regions of spectrotemporal modulation space related to voice pitch (reviewed in [85], see also [97]). The fact that nearby human dorsal premotor regions respond to auditory signals carrying rhythmic and pitch-related information indicates that strong neural signals flow from auditory regions to these premotor regions [101].

It is very likely that neural signals also flow in the opposite premotor-to-auditory direction, given that fine motor control based on auditory input requires rapid and precise bidirectional interactions between auditory and motor regions, i.e., both inverse and forward models. Indeed, in a recent study of speech processing combining intracranial recordings with mathematical simulations, Shamma and colleagues argued that motor-to-auditory projections (forward model) are essential for vocal learning and not just for the online control of learned vocalizations [54]. Motor-to-auditory projections are also likely important to the human brain’s ability to accurately predict the timing of musical beats [66].

In summary, both the parrot and the human vocal learning system have a dual pathway architecture, and in each case, the evolution of one of the pathways—the shell system in parrots and the laryngeal pitch control system in humans—seems potentially relevant to the capacity for BPS because of the way it enhances forebrain auditor-motor integration. Yet given that vocal learning pathways are involved in integrating complex auditory processing with *vocal* motor control, why would this lead to *nonvocal* movements in response to music? This question is addressed in the next section.

### From vocal learning to beat-synchronized nonvocal movements

Why would the evolution of strong forebrain auditory-motor integration in the service of learning *vocal* movements lead to auditory processing influencing *nonvocal* movements? Chakraborty and Jarvis [83] and Hickok and colleagues [85] both offer brief thoughts on this question. Below I quote their suggestions and propose an idea building on the work of both groups.

After describing the core and shell systems in parrots and suggesting that the shell system evolved via a brain pathway duplication of the core system, Chakraborty and Jarvis [83] write that “This dual system evolved early in the parrot lineage, and has lasted and expanded for millions of years in different species. In addition, changes in the regulation of some genes that may allow greater vocal–motor–auditory integration in vocal learning systems could have influenced changes in the surrounding motor areas to allow greater auditory–motor entrainment and synchronizing of body movements to the rhythm of music for dance in parrots.” As I understand it, the proposal is that changes in gene regulation associated with the evolution of the shell system fortuitously led to enhancement in the strength and/or temporal precision of neural communication between auditory and *nonvocal* premotor regions immediately adjacent to shell premotor regions [102]. Chakraborty and Jarvis suggest that this granted parrots a capacity for BPS. An appealing aspect of this idea is that it could help explain why parrots have the capacity for nonvocal beat synchronization even though they do not seem to use this ability in their natural behavior.

Hickok and colleagues propose a different idea [85]. They write “Perhaps rhythmic synchronization, the ability to synchronize movement to an auditory beat—a rare trait found only in species with complex vocal learning—is a necessary function enabling the coordination of the two proposed streams.” In other words, beat synchronization is an ability that grows out of the need to solve a coordination problem between two complex vocal control systems: a laryngeal pitch control system and a supralaryngeal articulatory system. This idea is interesting in light of the fact that parrots coordinate complex syrinx and tongue movements during learned vocalizations, and produce songs with a structure reminiscent of the phonological structure of speech [94].

Synthesizing the ideas of Chakraborty and Jarvis with those of Hickok and colleagues, I propose that the evolution of strong integration between auditory regions and vocal dorsal premotor regions in ancestral humans (via the laryngeal pitch control pathway) involved gene regulation changes which fortuitously enhanced the strength of neural connections between auditory and nonvocal dorsal premotor regions near the vocal dorsal premotor regions. (For recent evidence that the evolution of vocal learning in mammals is linked to gene regulation changes, see [103]). Below I specify a particular neural pathway I suspect was fortuitously strengthened in this way, e.g., via an increase in the number and/or myelination of axons in this pathway during brain development. While this proposal does not view the capacity for BPS as arising out of the need to coordinate two vocal control systems, it relies on Hickok and colleagues’ proposal of a distinct laryngeal pitch control pathway. The critical feature of this pathway is that it involves strong, temporally precise communication between auditory cortical regions and *dorsal* premotor cortex, reminiscent of the auditory—dorsal premotor communication involved in beat processing (Fig. 1) [63–65].

The exact white matter pathways linking auditory cortex and the laryngeal dorsal premotor region are not yet known, and may involve direct projections and/or projections that go through a third region such as sensorimotor area Spt (Sylvian parietal-temporal cortex) [104]. In keeping with the “Action Simulation for Auditory Prediction” (ASAP) hypothesis [71], I believe that the auditory—premotor connections supporting beat processing go via a dorsal auditory stream pathway linking auditory and dorsal motor planning regions via the parietal cortex [105]. The ASAP hypothesis is consistent with fMRI research indicating that beat perception (even in the absence of movement) engages inferior parietal regions near the angular gyrus [106], and with transcranial magnetic stimulation (TMS) research showing that transiently disrupting neural activity in the vicinity of angular gyrus selectively degrades beat perception but not single-interval timing [107].

**Fig. 4:**
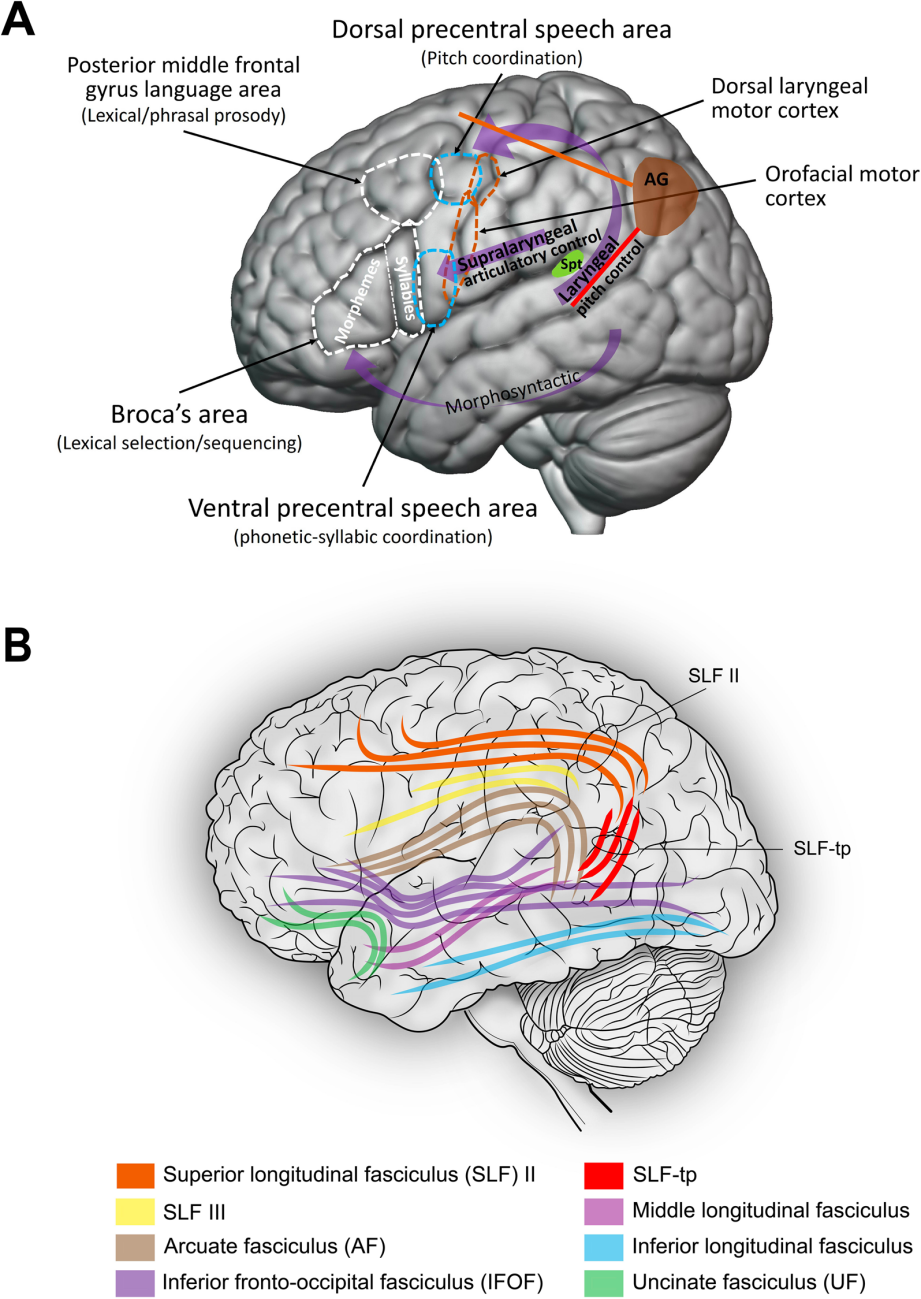
Cortical pathways for speech motor control and proposed dorsal stream pathway involved in beat-based processing. **A** Hickok et al. s dual speech coordination system model [85] (cf. Fig. 3) with the proposed dorsal stream pathway involved in beat processing superimposed [71]. This pathway is schematically shown as two lines connecting to inferior parietal cortex in the vicinity of angular gyrus (AG). The red line connects auditory regions in poterior superior temporal gyrus to this inferior parietal region, and the orange line connects this inferior parietal region to dorsal premotor regions. Area Spt (Sylvian parietal-temporal cortex), a sensorimotor region involved in both speech and music, is indicated by a small green circle for reference. (Locations of AG and Spt in this figure are approximate and were kindly provided by Greg Hickok, who notes that Spt is mostly located within the Sylvian fissure at its posterior-most extent and is difficult to see in a lateral reconstruction.) The red and orange lines in **A** are suggested to be part of known brain pathways shown in (B), based on a review of the neuroanatomy of language by Edward Chang and colleagues [109]. Specifically, the solid red line in (**A**) is proposed to be part of the temporo-parietal branch of the superior longitudinal fasciculus (SLF-tp, red lines in B), and the solid orange line in (**A**) is proposed to be part of the second branch of the superior longitudinal fasciculus (SLF-II, orange lines in **B**)

Figure 4A reproduces the dual speech coordination model of Hickok and colleagues from Fig. 3, but now also shows the approximate location of angular gyrus (AG, brown region) along with a red line schematically indicating proposed white matter connections between auditory regions and AG which were fortuitously made stronger and/or more temporally precise due to the evolution of the laryngeal pitch control pathway. Connections between angular gyrus and auditory regions in superior temporal gyrus are recognized in human neuroanatomy [108], where they are sometimes considered part of the posterior segment of the arcuate fasciculus, a tract also known as the temporo-parietal branch of the superior longitudinal fasciculus or SLF-tp, shown in Fig. 4B [109]. It is important to emphasize that I am not claiming that this entire fiber tract arose as a byproduct of the dorsal laryngeal pitch control pathway. The tract likely arose much earlier in brain evolution in the context of other functions, e.g., multimodal processing, since the angular gyrus receives auditory, visual, and somatosensory input [110]. The claim is that the strength and temporal precision of this pathway was fortuitously enhanced by gene regulation changes associated with the evolution/strengthening of the dorsal laryngeal pitch control pathway.

Note that the red line in Fig. 4A connecting auditory regions and AG is not meant to indicate precise localization in terms of neural connections and is simply a schematic indicating connections between these regions. Higher-order (non-primary) regions in posterior superior temporal gyrus (pSTG) would be good candidates for the auditory regions since they contain neural populations selective for music [111] and are in regions that would be part of the dorsal processing stream. Furthermore, auditory neurons in posterior higher-order auditory regions have significantly shorter latencies than neurons in anterior higher-order regions [112], making them well-suited to temporally-precise temporal interactions with other brain regions. Finally, twin studies indicate that the structure of the posterior arcuate fasciculus has a substantial genetic influence [113]. Thus evolutionary changes in genes regulating axonal numbers, diameter, myelination, or targeting in this tract could have influenced neural communication between nonprimary auditory regions in pSTG and inferior parietal regions in the vicinity of the angular gyrus.

Figure 4A also shows a schematic white matter connection (solid orange line) between angular gyrus and dorsal premotor regions via a brain pathway known to exist in humans and other primates [114], namely branch 2 of the superior longitudinal fasciculus (SLF II), a tract shown in Fig. 4B. My proposal is that together the red and orange connections in Fig. 4A, which link auditory cortical regions to dorsal premotor regions via the angular gyrus, created a path for strong and temporally precise bidirectional communication between auditory and nonvocal premotor cortex. This would grant ancestral humans a capacity for BPS without natural selection for this ability. Notably, diverse and complex rhythmic nonvocal movements to music would be a plausible consequence of connecting auditory rhythmic processing to dorsal premotor regions involved in control of regions such as the hands, arms, and feet. Such premotor regions lie above the dorsal precentral speech area, and auditory input to these regions would scaffold clapping, stepping, and other nonvocal rhythmic movements in response to beat-based auditory rhythms.

Of course, such ancestral humans would need a source of beat-based rhythms to trigger such rhythmic movements. For parrots today this source is human music, but for ancestral humans the rhythmic sounds must have been simpler, perhaps consisting of rhythmic vocalizations or drumming. Since rhythmic sounds are made by modern humans and by chimpanzees (e.g., chanting in humans, pant hooting in chimpanzees, and drumming in both species [115–117]), a last common ancestor would likely have been able to produce periodic auditory rhythms without difficulty. Indeed, a zoo chimpanzee has been observed to spontaneously drum a rhythmic pattern with episodes of periodic structure over the course of several minutes, consisting of hundreds of percussive events [116]. This indicates that voluntarily *making* sustained periodic acoustic rhythms does not depend on advanced vocal learning. (Indeed, even insects such as crickets can make periodic acoustic rhythms.) The current paper argues that without the evolution of advanced vocal learning, however, periodic sonic rhythms would never spark spontaneous BPS.

While beyond the scope of the current paper, it is worth noting that the fortuitous auditory-nonvocal premotor connections supporting BPS in ancestral humans may have been strengthened by natural selection due to the psychological and social effects of BPS in group settings. This idea is presented in more detail in Patel (2021) [41], where I proposed that such strengthening occurred via a process of gene-culture coevolution. Such strengthening may have involved an increase in the number and/or myelination of neural fibers in the white matter pathways schematically represented by the red and orange lines in Fig. 4A [118]. These stronger pathways could support the adult human ability to sustain stable BPS to music for several minutes, in contrast to parrots, who do not seem able to sustain accurate BPS for long periods. Perhaps the limited BPS abilities of parrots resemble the capacities of human ancestors prior to natural selection for BPS abilities.

### Proclivity and species-specificity in beat-synchronized movement to music

The focus of this paper so far has been on the capacity for BPS, arguing that it first emerged as a byproduct of an advanced vocal learning system. Yet why would ancestral humans, like some parrots today, *spontaneously* develop BPS in response to complex beat-based rhythms? Simply having a fortuitous capacity for a behavior does not automatically entail a proclivity for the behavior. For example, internet videos show that some dogs can learn to walk on two legs for prolonged periods (e.g., after forelimb injury), but no dogs spontaneously do this. Thus even if future work shows that many species can learn to synchronize movements to musical beats based on formal training (cf. Box 1), it remains to be explained why predictive and tempo-flexible beat synchronization to music emerges without such training only in humans and parrots.

I propose that an advanced vocal learning system is one important factor underlying the spontaneous emergence of BPS in animals raised around beat-based rhythms. I hasten to add that other factors are almost certainly crucial, and it is likely the confluence of advanced vocal learning with these other factors which drives the emergence of BPS without formal training. One such factor could be the ability to imitate nonvocal movements [74, 119], which is present in humans and parrots [120] and has been theorized to be a key capacity underlying the evolution of human dance [121]. Another factor, also present in humans and parrots, could be a craving for social interaction and a strong sensitivity to social reward [122]. Indeed, Snowball and other pet parrots that move rhythmically to music often get attention and praise from their owners for this behavior. Notably, however, motor imitation abilities and a strong sensitivity to social reward cannot alone prompt BPS to emerge. Human-reared chimpanzees are adept at visual imitation and seek social interaction with their caretakers [32, 33], and dogs bond with their owners and are highly sensitive to social reward such as praise [123], but neither of these species has been observed to spontaneously engage in BPS when reared by humans.

Why would an advanced vocal learning system facilitate the spontaneous emergence of BPS? Such a system provides an intrinsic motivation to learn the structure of complex auditory sequences. For advanced vocal learners, this motivation is needed to learn the structure of their natural communication system [124]. Like humans, parrots seem to retain this motivation throughout life, as shown by the fact that adult parrots can spontaneously learn to produce new sounds that they hear (e.g., phrases from a new human caretaker) [92]. Cognitive research suggests that learning sound sequence structure involves implicitly predicting upcoming sounds and comparing those predictions to incoming sensory data in order to update internal models of sequence structure [125]. When such a learning system is presented with sequences with periodic beats, predictions of beat timing could naturally begin to occur. If nonvocal motor planning regions are involved in making such periodic temporal predictions, as suggested by numerous researchers [66], and if these motor regions have strong reciprocal connections with auditory regions, this could predispose spontaneous movement to the beat of music during ontogeny. A tendency to visually imitate others and to experience reward in response to positive social attention could interact with this predisposition to amplify BPS behavior [126].

The idea that spontaneous BPS to music depends on a convergence of several distinct factors (among which advanced vocal learning is just one) might help address why this behavior has not been observed in some highly talented vocal learning species. For example, mynah birds, like parrots, are sometimes kept as pets and can mimic speech with remarkable fidelity [127]. Mockingbirds and brown thrashers are also excellent vocal mimics, able to imitate the songs of many other birds. Given these advanced vocal learning skills, it seems possible that these birds, like parrots, have evolved an unusually complex vocal learning system. It is currently unknown if these birds have evolved something analogous to the core/shell song system of parrots. If they have, then by the current paper’s logic this should involve enhanced forebrain audiomotor integration and scaffold fortuitous BPS abilities. Yet there are no reports of BPS developing spontaneously in pet mynahs raised around beat-based human music. (Mockingbirds and brown thrashers are generally not kept as pets, so their response to being raised around beat-based music is not known.) Perhaps this is because mynahs lack a capacity for nonvocal movement imitation. BPS has also never been reported in pet ravens, even though they can imitate speech (T. Fitch, personal communication) and nonvocal movements [128]. Perhaps this is because pet ravens are not as sensitive to social reward (attention and praise) from humans as pet parrots are. The larger point is that a key issue for future research on the evolution of dance is identifying the necessary and sufficient set of neurological capacities leading to the spontaneous emergence of BPS to beat-based rhythms during ontogeny.

### Predictions and future directions

The proposal outlined in this paper leads to specific predictions about the neuroanatomy of beat-based rhythmic processing. Specifically, it predicts that “beat deaf” individuals who struggle to perceive and/or move in synchrony with musical beats [129, 130] will differ from proficient beat-synchronizers in the structure of either or both of two specific brain pathways (e.g., showing reduced volume or organization of fibers). These are dorsal stream pathways that reciprocally link cortical auditory and dorsal premotor regions via inferior parietal cortex, schematically shown by orange and red lines in Fig. 4A. One of these pathways overlaps with the posterior segment of the arcuate fasciculus, also known as the temporo-parietal branch of the superior longitudinal fasciculus (SLF-tp in Fig. 4B). The other is the second branch of superior longitudinal fasciculus (SLF II in Fig. 4B). Beyond group-level comparisons between beat-deaf and non beat-deaf individuals, it would also be interesting to examine how individual differences in the structure of these tracts relate to individual differences in musical beat perception or synchronization abilities.

The current proposal also motivates cross-species research on the structure of the above brain pathways in humans vs. nonhuman primates, since no nonhuman primates are known to spontaneously develop BPS when raised around beat-based music. In line with an earlier paper [41], the current paper predicts auditory-parietal connections via SLF-tp are much more strongly developed in humans vs. nonhuman primates. It would also be interesting to see if parietal to dorsal premotor connections via SLF II are stronger in humans vs. other primates, especially in chimpanzees in which SLF II is a well-developed tract [131, 132].

In addition to these cross-species studies the current proposal also motivates increased attention to the role of inferior parietal regions in beat-based rhythmic processing in humans. Several human neuroimaging studies have implicated inferior parietal regions in beat or dance processing (including perceptual studies with no overt movement) [69, 106, 133], and TMS to angular gyrus has been shown to disrupt purely perceptual beat processing [107]. However, the specific contributions of inferior parietal cortex to beat processing and dance remain to be elucidated. As a region known to be involved in sensorimotor integration, it would be particularly interesting to determine if inferior parietal cortex plays a key role in integrating bottom-up signals about event timing from auditory regions with top-down predictive signals from premotor regions during beat and dance processing.

Finally, since the current proposal views beat-based synchronization to music as fortuitously originating from the evolution of an advanced vocal learning system, it motivates examining the genetic, neural, and cognitive overlap of beat processing and phonological processing (i.e., processing of the sound structure of language), especially the role that SLF-tp plays in both of these abilities in young children [134]. The proposal thus aligns with the recent Musical Abilities, Pleiotropy, Language, and Environment (MAPLE) framework for music-language links in the human mind [135].

## Conclusions

Comparative research on beat-based movement to music in humans and parrots can shed light on the evolutionary origins of human dance. Pet parrots appear to be the only nonhuman animals that spontaneously develop predictive and tempo-flexible synchronization to musical beats, even though parrots and humans are separated by hundreds of millions of years of evolution. By synthesizing recent neural work on parrot vocal learning and on human speech motor control, this paper proposes how and why the evolution of advanced vocal learning in parrots and ancestral humans fortuitously led to the capacity and proclivity to synchronize nonvocal movements to beat-based auditory rhythms. This proposal specifies an auditory-parietal cortical pathway in human brains that fortuitously changed in ways that support predictive and tempo flexible beat synchronization, leading to testable predictions and to suggestions for future research.

## Commentaries and response

This article is published along with four commentaries and a response by the author.

### Commentaries

Hickok, G. The “coordination conjecture” as an alternative to Patel’s fortuitous enhancement hypothesis for the relation between vocal learning and beat-based dancing. [136]

Penhune, VB. How different are the differences? A commentary on the paper “Beat-based dancing to music has evolutionary foundations in vocal learning.” [137]

Schmidt MF, Kaplan M. Toward a comparative understanding of beat perception and synchronization. [138]

Theofanopoulou, C. Tapping into the vocal learning and rhythmic synchronization hypothesis. [139]

### Response

Patel, AD. Response to commentaries by Schmidt and Kaplan, Penhune, Hickok, and Theofanopoulou on “Beat-based dancing to music has evolutionary foundations in advanced vocal learning.” [140]

## Data Availability

Not applicable.

## Abbreviations

AAC: Central nucleus of the anterior arcopallium in the parrot forebrain, part of the core song system, analogous to forebrain region RA in songbirds
AG: Angular gyrus in humans
BPS: Beat perception and synchronization
fMRI: Functional magnetic resonance imaging
HVC: High Vocal Center, a songbird premotor nucleus reciprocally connected to forebrain auditory nuclei and necessary for the learning and production of song
MRI: Magnetic resonance imaging
RA: Robust nucleus of the arcopallium in songbirds, a forebrain motor nucleus involved in controlling the songbird sound production organ (syrinx)
pSTG: Posterior superior temporal gyrus in humans
TMS: Transcranial magnetic stimulation
SLF II: Superior-longitudinal fasciculus, second branch in humans
SLF-tp: Superior-longitudinal fasciculus, temporo-parietal branch in humans
Spt: Sylvian parietal-temporal cortex in humans
STRF: Spectrotemporal receptive field
VLRSH: Vocal learning and rhythmic synchronization hypothesis
XII: Brainstem hypoglossal nucleus in parrots and songbirds (labelled XII because it is innervated by the 12th cranial nerve)

## Footnotes

1 Elephants in sanctuaries have been shown to synchronize rhythmic movements to an auditory beat [26] but as noted by the lead author of that study, it is not clear if this behavior was spontaneous or trained, and tempo flexibility was not tested [39]. Elephants are vocal learners, but their exact degree of vocal flexibility is not yet known [40].
